# Sea level rise and the evolution of aggression on islands

**DOI:** 10.1016/j.isci.2024.111236

**Published:** 2024-10-22

**Authors:** Kenneth F. Rijsdijk, Jasper C. Croll, Julian P. Hume, Anwar Janoo, Robin Aguilée, Johannes De Groeve, Rosemarie Kentie, Menno Schilthuizen, Ben H. Warren, Leon P.A.M. Claessens

**Affiliations:** 1 Institute for Biodiversity and Ecosystem Dynamics (IBED), TCE, University of Amsterdam, P.O. Box 94240, Amsterdam 1090 GE, The Netherlands; 2 Bird Group, Department of Life Sciences, Natural History Museum, Akeman St, Herts, Tring HP23 6AP, UK; 3 National Heritage Fund, Fon Sing Building, Edith Cavell St., Port Louis, Mauritius; 4 Centre de Recherche sur la Biodiversité et l'Environnement (CRBE; UMR5300), Université de Toulouse, CNRS, IRD, Toulouse INP, Université Toulouse 3 - Paul Sabatier (UT3), Toulouse, France; 5 Royal Netherlands Institute for Sea Research (NIOZ), P.O. Box 59, Den Burg, Texel 1790 AB, The Netherlands; 6 Naturalis Biodiversity Center, P.O. Box 9517, Leiden 2300 RA, The Netherlands; 7 Institut de Systématique, Evolution, Biodiversité (ISYEB), Muséum National d’Histoire Naturelle, CNRS, Sorbonne Université, EPHE, UA, Paris, France; 8 Maastricht Science Programme, Faculty of Science and Engineering, University of Maastricht, P.O. Box 616, Maastricht 6200 MD, The Netherlands

**Keywords:** Ecology, Zoology, Evolutionary biology

## Abstract

Why aggressive traits evolve in some species but not in others is poorly understood. We modeled the population dynamics of the extinct Mauritius dodo and Rodrigues solitaire to examine divergent pathways in the evolution of aggression. Whereas the dodo conformed to island syndrome predictions of tameness, its sister-taxon the solitaire evolved strong sexual dimorphism and aggressive traits. We computed rates of change in island size from sea level modeling and connected island size change to population dynamics by integrating a Hawk-Dove game theory model for territory competition with a population model. We find that the rapid rate of decrease in island size likely was an important trigger for the onset of aggressive behavior and that aggressive behavior becomes fixed if a tipping point is reached where island size falls below a critical threshold.

## Highlights

• The solitaire evolved aggressive traits while the closely related dodo did not
• We suggest that this disparity emerged from differences in island size changes
• We explore this by linking a sea level model, game theory and population dynamics
• In the model, aggressiveness becomes fixed if island size crosses a tipping point

## Graphical abstract

**Figure:**
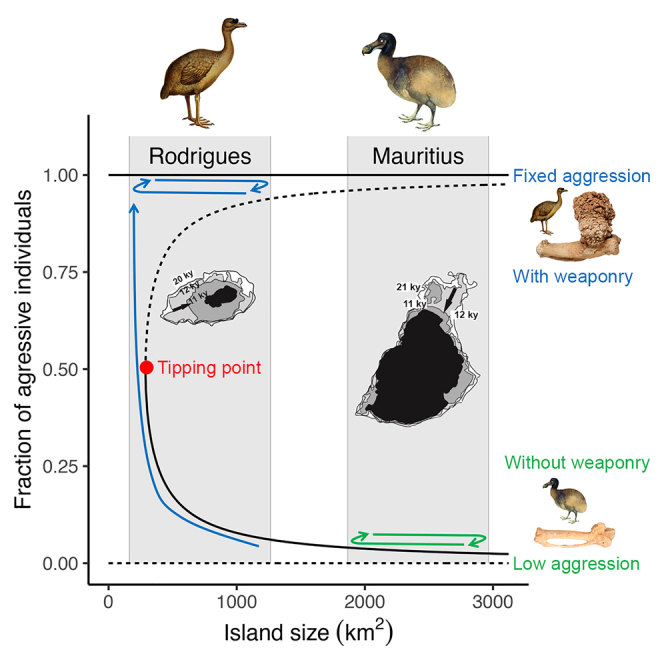

## Introduction

Aggressive traits and strategies help mediate dominance and success in group defenses or in accessing critical resources including food, mates, and nesting sites. Body size, fighting ability, and weaponry can be decisive factors.^1^ For example, increased competition due to seasonally reduced availability of resources such as food and nesting sites affects hormone levels and has been shown to trigger seasonal aggressive behavior.^2^^,^^3^^,^^4^^,^^5^^,^^6^^,^^7^ Rates of aggression are often highest among the densest populations^1^ and it is a general assumption that aggressive traits evolve when the individual benefits of aggressive behavior (such as access to limited resources) outweigh the costs of aggressive behavior (such as risk of injury, loss of time, and energy).^8^^,^^9^^,^^10^ However, it is not clear how the emergence and fixation of aggressive behavioral traits takes place in environments where the effects of habitat size and quality experience temporal variation.^5^ On islands, species typically become more tame, as part of a complex of changes often described as *island syndrome*,^11^^,^^12^^,^^13^^,^^14^^,^^15^ although the reverse has been observed with small or fluctuating population sizes.^16^^,^^17^

**Figure 1:**
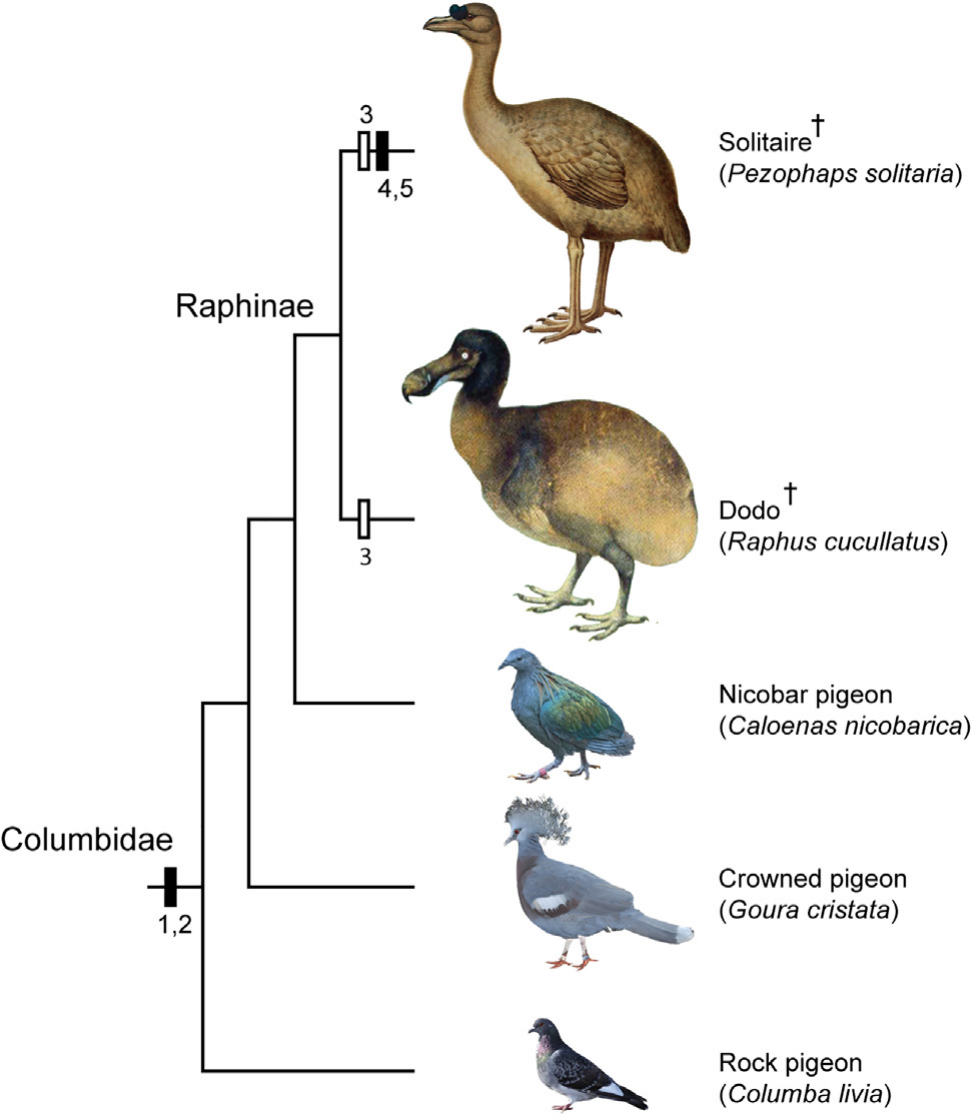
Phylogeny of Columbidae showing the placement of the extinct Raphinae The dodo and the solitaire are closely related to the Nicobar and crowned pigeons. Characters: 1. magnitude of skeletal sexual size dimorphy 5–10%; 2. Wing slapping used in antagonistic interactions; 3. Loss of flight, evolved independently in the dodo and solitaire; 4. Extreme skeletal size sexual dimorphism, >30% in limb skeleton; 5. Skeletal specializations for combat for wing combat and display.

**Figure 2:**
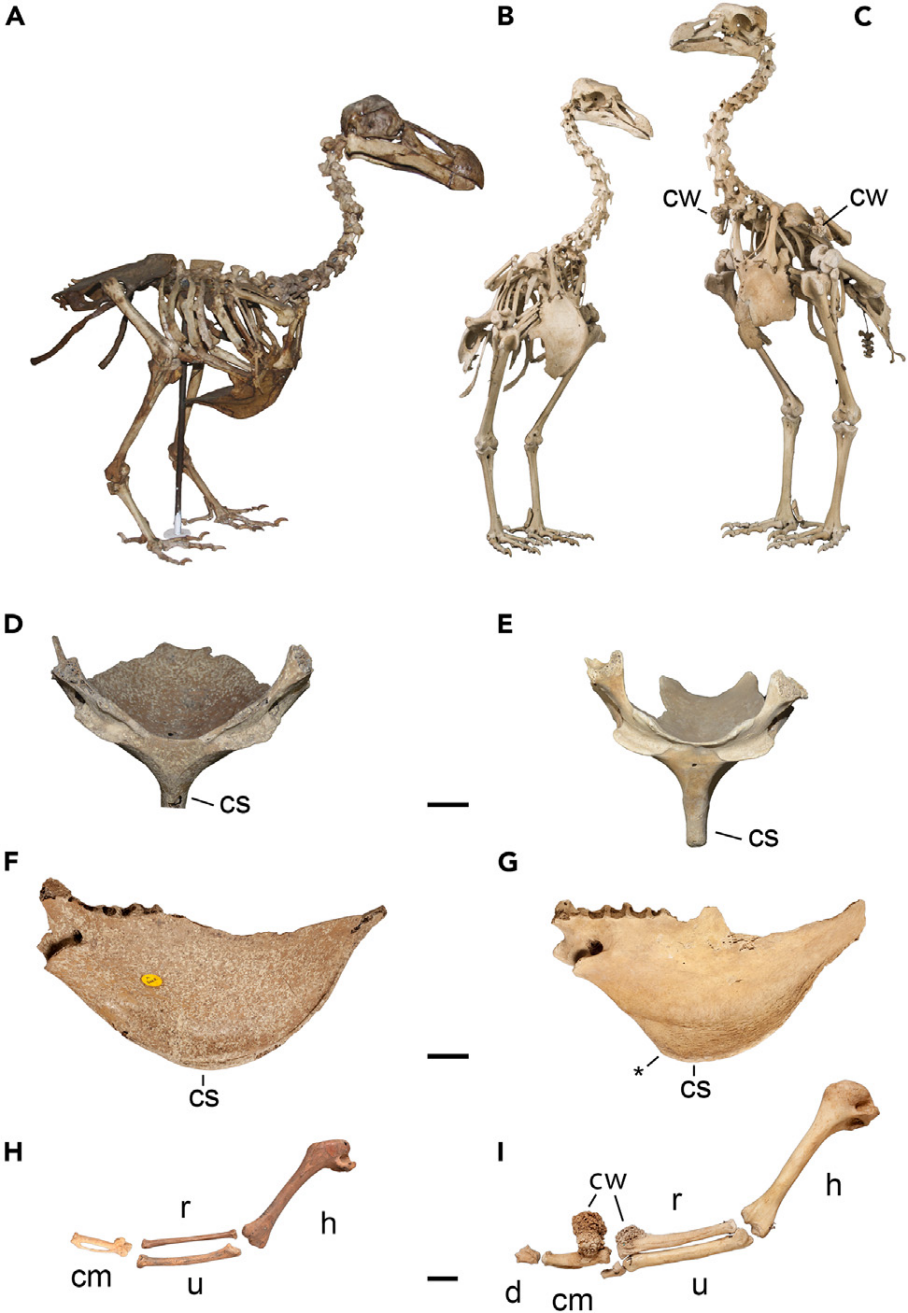
Comparison of the skeletal anatomy of the dodo (A, D, F, H) and solitaire (B, C, E, G, I) Dodo skeleton (A) in right lateral view, sex unknown, comparable to relatively larger female or smaller male. Female (B) and male (C) solitaire skeletons in oblique anterior view. Dodo sternum in anterior (D) and left lateral (F) view, and solitaire sternum in anterior (E) and left lateral (G) view. Dodo (H) and solitaire (I) wing skeleton. The dodo (A) does not show any wing weaponry and only modest sexual dimorphy. Extreme skeletal sexual size dimorphy, especially in hindlimb length, and wing weaponry, can be observed in the solitaire, with the male being much taller and equipped with pronounced wing weaponry (C and I). The female solitaire (B) is smaller and does not exhibit wing weaponry of a similar magnitude. The solitaire has a deeper sternal keel (E and G) with a distinct anterior apex (G, asterisk). The bony growths on the wing skeleton can become very large and extend from the distal radius, proximal carpometacarpus and even wing digits (I). Specimens: (A) MI Port Louis Thirioux dodo^21^; (B) RCSHM/Aves 706; (C) RCSHM/Aves 707; (D and F) NHMUK PVA3622, (E and G) NHMUK PVA1377; (H) NHMUK PVAu/r, (I) NHMUK PVA1441. Abbreviations: cm, carpometacarpus; cs, sternal keel (carina sterni); cw, carpal weaponry; d, wing digit (phalanx proximales digiti majoris); h, humerus; r, radius; ulna; asterisk denotes apex carina sterni, visible in the solitaire. Scale bar (D–I) is 2 cm.

Here, we present the remarkable case of two sister species of extinct giant flightless pigeons, the dodo (*Raphus cucullatus*, Linnaeus, 1758) and the solitaire (*Pezophaps solitaria*, Gmelin, 1789) (Figure 1), which were endemic to the neighboring Mascarene Islands of Mauritius and Rodrigues, situated approximately 600 km apart in the southwestern Indian Ocean.^18^^,^^19^ Conforming with *island syndrome* predictions,^11^^,^^12^ the behavior of the dodo and other birds on Mauritius was described as naive and without fear of humans, traits that are frequently implicated in the dodo’s demise.^18^^,^^19^^,^^20^^,^^21^ In contrast, the solitaire was described as highly territorial and aggressive when defending its territory, exhibited pronounced sexual dimorphism, and possessed skeletal adaptations for intraspecific combat, including large bony knobs on the wrist that were used in violent wing claps (Figures 2A–2I).^22^^,^^23^

It is intriguing why only one of the two largest extinct flightless pigeons, the solitaire of Rodrigues, evolved intraspecific aggressive behavior and corresponding phenotypic traits, whereas its closest relative on neighboring Mauritius, the dodo, did not. The divergent outcomes in the evolution of aggressive behavior between the two largest extinct pigeons, which lived in a similar climatic and ecological setting, may provide important clues in the evolution of aggression through habitat changes caused by sea level fluctuations.^24^^,^^25^^,^^26^ Links have been suggested between the evolution of aggressiveness in the solitaire and island size reduction due to sea level change. Rodrigues is much smaller than Mauritius, with presumed higher seasonal limitations in resource availability, and underwent a more pronounced decrease in size at the end of the Pleistocene when sea level rose.^22^^,^^27^ However, these hypotheses for geophysical drivers of the evolution of aggressiveness have not been explored further or substantiated, despite the potential of the Mauritius-Rodrigues system to study the outcome of differences in degrees of habitat loss by Pleistocene sea level rise under similar climatic and ecological conditions.

Here, we investigate how the dynamics of the island size could affect the emergence and dynamics of aggressive behavior. We first examine how the dynamics of island size reduction can result in the emergence of aggressive anatomical traits and behavior. We then study how aggressiveness spreads throughout the population once it emerged. To do so, we integrate results from a geophysical sea level model to quantify the area changes of these Mascarene islands in a novel population dynamic modeling framework, in which we connect a classic Hawk-Dove game for territory competition with individual territory competition and reproduction rates.

## Results and discussion

### Anatomy

The anatomy of the dodo and solitaire differed substantially with respect to overall body shape, the degree of sexual dimorphism, and adaptations for (intraspecific) combat (Figures 2A–2I). The anatomical weaponry of the solitaire included a callus-like exostosis or carpal knob on the proximal end of the carpometacarpus that was described as a “musket ball” by observers who saw the bird alive (Figures 2C and 2I).^23^^,^^28^ These knobs were covered in a thickened, cartilaginous or keratinous integument measuring up to 5 cm in diameter.^22^ They were largest in males but also present in females.^22^ Pigeons use wing slapping in territorial defense^29^^,^^30^ and therefore the development of wing weaponry in solitaires is a potential result of aggressive territorial behavior. Indeed, historical reports indicate that the carpal knobs were knocked together or drummed on the body to audibly delimit a breeding territory and to deter rival birds, and both sexes were reported to have fought opponents with violent wing claps.^22^^,^^23^^,^^28^ Multiple examples of healed fractures in fossil solitaire bones highlight the use of its wing weaponry in fights, and the risks and cost associated with this aggressive behavior, whereas healed fractures in the dodo are extremely rare and anatomical adaptations for combat in the wing skeleton are absent (Table 1).^22^ The sternum of the flightless solitaire retained a large keel^22^ and a distinct anterior apex that would have aided muscular attachment to swing the weaponized wings forward (Figures 2D–2G). The dodo, in contrast, had a more vestigial sternal keel, which would have limited the force of a swinging motion (see also in the studies by Hume et al. and Claessens L.P.A.M et al.^21^^,^^22^; Figures 2G and 2I).

**Table 1:** Healed fractured wing bones observed in the solitaire

Skeletal element	Right	Left	Total
Coracoid	1 (18)	1 (27)	2 (45)
Humerus	2 (12)	0 (11)	2 (23)
Radius	0 (32)	1 (31)	1 (63)
Ulna	7 (45)	7 (54)	14 (99)
Carpometacarpus	2 (9)	0 (11)	2 (20)

Number of healed fractured wing bones observed in the solitaire as a fraction of the total number of skeletal elements examined. Number of broken bones listed with total number of bones examined in parentheses. Data reported separately for right and left side of the body. Note that most breaks occur in the ulna, one of the main striking wing elements where the carpal knob is located. Only a single broken dodo bone, a coracoid, has been observed during our study.

### Geology

Oceanic island sizes have fluctuated globally with sea level fluctuations, with different islands showing different responses in area change over individual glacial-interglacial cycles.^24^^,^^25^^,^^26^^,^^31^ We used a geophysical-based sea level model to compute absolute changes in island size for Mauritius and Rodrigues^26^^,^^31^ (see STAR Methods sea level modeling).

Mauritius and Rodrigues are neighboring volcanic islands that were formed by activity of the Réunion hotspot. The larger island of Mauritius (1852 km^2^) emerged above sea level approximately 10-8 My ago and has remained a persistent island since.^32^ The geological history of the smaller island of Rodrigues (108 km^2^) is more complex and relevant for the modeling. Rodrigues Island is located on the Rodrigues Ridge,^33^^,^^34^ an east-west running linear volcanic structure of approximately 760 km in length.^34^ The subaquatic base of Rodrigues Island formed close to the time of formation of Mauritius. Approximately10-8 My ago, Rodrigues Ridge may have been a chain of volcanic islands that were subsequently reduced by marine planation and formed guyots.^34^ At the location of Rodrigues Island, the ridge forms a guyot topped with an elliptical submarine wave-cut platform at approximately 50–60 m below sea level.^34^ This platform is covered by a thick bed of reef corals that were deposited in the Pliocene under conditions of prevailing high sea level stands that were up to 20 m higher than present-day conditions.^25^^,^^34^^,^^35^^,^^36^^,^^37^ Radiometric dates on lavas suggest that modern Rodrigues Island, which has a current height of 398 m, formed as an island 1.6 My ago as the result of a new phase of volcanic activity, building further upon the elliptical-shaped guyot.^33^^,^^34^ However, prior to that, the coral reef platform may at times have been subaerial and supported low elevation shoals or atolls, a hypothesis that is supported by the depth of genetic divergence and corresponding date estimates for Rodrigues-endemic invertebrate clades that exceed 1.6 My.^38^^,^^39^^,^^40^ Only after the formation of Rodrigues as a persistent island, at least 1.6 My ago, could loss of flight take place in the solitaire’s ancestral lineage.

**Figure 3:**
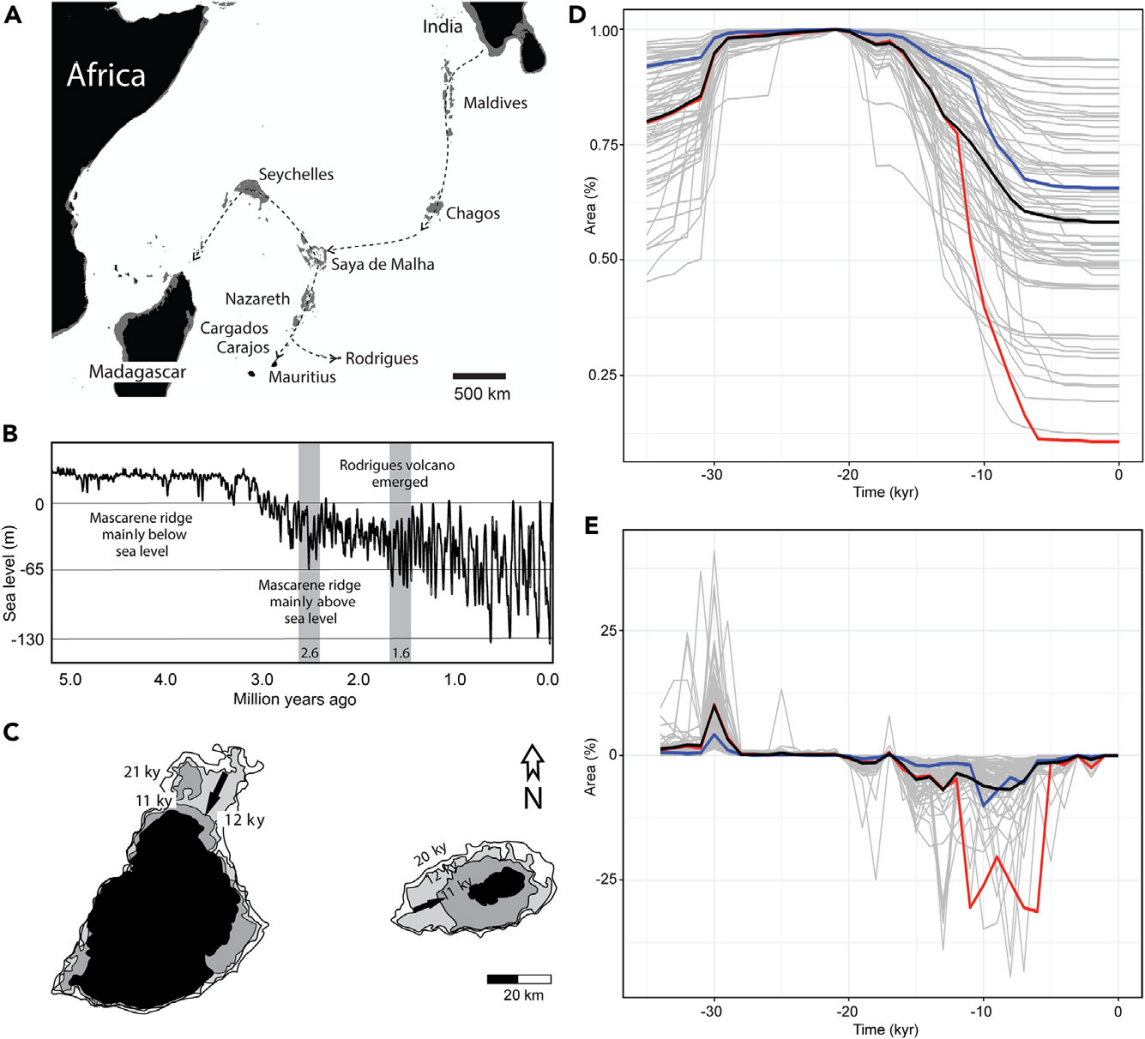
Sea level fluctuation and island size (A) The extent of the Maldives, Lacadives and Mascarenes. Present-day coastline and landmass shown in black. Paleo-extent of the plateaus during the glacial maximum lowstand shown in gray. Inset boxes show Mauritius and Rodrigues and their maximum extent during glacial times when sea levels were lower than 60 m BP. Arrows indicate the likely routes taken by the ancestor of the dodo and solitaire from India to Mauritius and Rodrigues. (B) Sea level change over the last 5 My. With the onset of the Pleistocene 2.6 My ago, the Mascarenes, Chagos, and Maladive ridges emerged and formed a persistent pathway to Mauritius and Rodrigues. (C) Mauritius (left) and Rodrigues (right). Present-day coastline and landmass shown in black, the extents of coastlines and landmasses at past periods shown in shades of gray. The shaded periods 11–10 ky BP at Mauritius, and 12–11 ky BP at Rodrigues, represent the periods of largest and fastest coastal contractions. The black arrows indicate the largest magnitude in coastal retreat, assuming gradual flooding over 1000 years. (D) Percentage of areas relative to maximum area achieved during the Last Glacial Maximum (LGM; 24–20 ky BP) of 68 volcanic islands over time. Excluded are islands <100 km^2^, fragmented islands, atolls, and non-volcanic islands. Mean relative area percentages (black line). After the LGM islands lost on average about 40% of the LGM maximum areas. Mauritius (blue line) lost about 30% of its LGM surface. Rodrigues (red line) stands out as the island that lost the largest proportion of area (90%) of all. (E) Percentage area change of 68 volcanic islands per time step of 1000 years. Black line is the mean area change for all islands. After the LGM, the maximum mean area reduction rate is about 7% per ky achieved at 14 ky and 8 ky BP. Mauritius (blue line) reaches its highest reduction rates between 11 and 6 ky BP. A maximum rate higher than 10% at 10 ky BP is achieved. Rodrigues (red line) stands out as an island with its highest reduction rates exceeding 30% per ky between 11 and 6 ky ago. The period of high area reduction rates is also one of the longest in duration (6 ky).

Our sea level modeling data show that the carbonate platform of Rodrigues Ridge became cyclically exposed in phase with the glacial cycles with the onset of the Quaternary ice ages (Figures 3A and 3B). The strong degree of land area reduction experienced by Rodrigues Island since its formation is exceptional compared to other volcanic islands, including Mauritius (Figures 3B–3E). It included pulses with land area losses of approximately 90% over a period of only 20 thousand years (ky) due to a 135 m postglacial sea rise in this time frame (Figures 3B–3E). However, the more extreme pulses of contracted state of Rodrigues lasted relatively briefly, less than 10% of the time, cumulatively, compared to Rodrigues island’s expanded state during glacial low sea level stands, which covered more than 900,000 of the last million years (Figures 3B, 3C, and 3E).

### Modeling framework

To explore the emergence and fixation of aggressive territory behavior in the dodo and solitaire we used a novel modeling approach in which we integrated a Hawk-Dove game model^8^^,^^9^ for territory competition into a population model. Our aim is to develop a model that could help to understand the difference in aggressiveness between dodos and solitaires, while keeping the model as simple as possible. In the model, we separated the costs and benefits of the Hawk-Dove game and linked these costs and benefits to specific mortality and reproduction rates of individuals. This approach was proposed by Argasinski and Broom^41^ and is widely used in the development of game theory, but as far as we know our study is the first time this model is linked to changes in habitat. In general, the dynamics of the population is determined by the birth and death of individuals. For simplicity we assume that all individuals have the same, constant, death rate. Individuals only reproduce when they obtain a territory. During the competition for territories, birds adopted either an aggressive (Hawk) strategy, or a non-aggressive (Dove) strategy. Individuals with the same strategy had equal chance to win the competition, while non-aggressive individuals would avoid fighting at all costs and give up their territories to aggressive individuals. This classic pay-off scheme represents the benefits from the Hawk-Dove game and directly determines the fraction of territories obtained by aggressive and non-aggressive individuals. In addition, only aggressive individuals have a risk to obtain an injury in a fight with another aggressive individual, representing the costs for the Hawk-Dove game. Only individuals that successfully obtained a territory and remained unharmed, were able to reproduce, while harmed individuals and individuals without a territory would not reproduce. In our model, territories are redistributed every year and individuals without a territory in the previous year or individuals previously wounded (and now healed) are allowed to compete again in the following year. In addition, we assume that individuals would adopt the same strategy (non-aggressive or aggressive) as their parents, either through learning or genetic heritability.

We used the model to simulate the population dynamics of aggressive and non-aggressive individuals under conditions of changing island surface areas. In the model, the dynamics of the population is determined by the mortality rate, the reproduction rate, the competition injury risk, and the number of available territories. The mortality rate was estimated based an average dodo and solitaire lifespan of 30 years^42^ Solitaire and dodo clutch size are modeled as one egg per year based on contemporary observations.^19^^,^^42^ This implies that the reproduction rate lies somewhere between zero and one depending on chick survival. The birth rate and injury risk were set to match expected population densities. We used bifurcation analysis to confirm that the parameter values affect the quantitative results, but not affect the mechanistic patterns determining the population dynamics (see STAR Methods Population Dynamics Modelling). The number of available territories was directly determined by the size of the island based on the described average territory size 0.104 km^2^ per territory. We used the island sizes from 120 ky ago until present calculated with our coastline reconstruction model. The dynamics during this period is representative of the area dynamics over glacial interglacial cycles during the last million years, when the amplitude and periodicity were comparable.^25^^,^^31^ It is important to note that the island size only determines the number of territories on the island and does not influence the behavior of individuals directly. Changes in population density and the balance between non-aggressive and aggressive individuals thus completely emerge from the ecological dynamics of the model.

### Emergence of aggression

Before aggressive behavior can spread through a population, it needs to emerge in a small number of individuals. We hypothesize that rapid contractions in island size could result in peaks in competitive pressure, which could trigger the emergence of aggressive behavior. If the dynamics of the island size change would have been slow compared to the generational lifespan of the dodo and solitaire (estimated at approximately 30 years^42^), we would expect population dynamics to more closely track the equilibrium state of the population. However, our coastline modeling shows that, for example, between 12 and 11 ky BP an extremely high rate of area loss occurred on Rodrigues, which amounted to a reduction of more than 40% (Figure 3E). Land area loss for Mauritius was much less pronounced over this period, with a reduction of approximately 10% (Figure 3E). The land loss rates for Rodrigues are among the highest recorded for oceanic volcanic islands (Figure 3E) and, in the Indian Ocean, is only exceeded by that experienced by the continental islands of the Seychelles.^26^ The maximum inland coastline migration rate during this time was approximately 10 m per year, amounting to approximately 190 m over the circa 30-year life span of solitaires.

To examine our hypothesis, we used our model to simulate the dynamics of a population with only non-aggressive individuals during a glacial cycle (Methods Population Dynamics Modelling A. Non-Aggressive Individuals). With a constant island size, the population size would be at a carrying capacity, which is dictated by the mortality rate and the number of territories. We analytically calculated that the population density at equilibrium is between 128,000 individuals (glacial maximum) and 80,000 individuals (interglacial minimum) for Mauritius, and between 54,600 (glacial maximum) and 7,000 (interglacial minimum) for Rodrigues. Although this is likely an overestimation of the number of individuals due to an overestimation of the number of territories (see STAR Methods Population Dynamics Modelling A. Non-Aggressive Individuals), it highlights the differences in the population size of dodos and solitaires, respectively.

**Figure 4:**
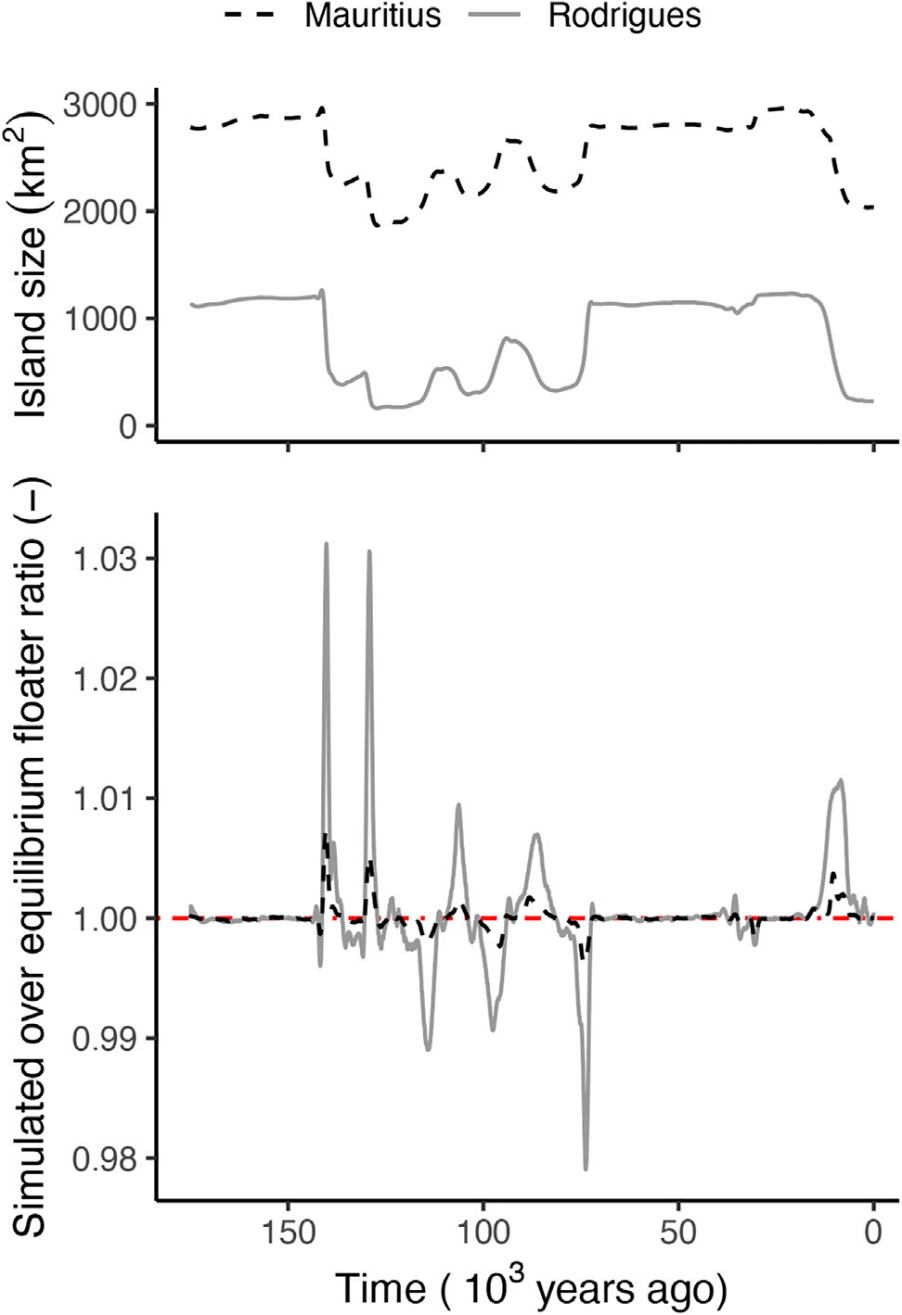
Estimate of change in competition due to changes in island size over an interglacial and glacial period The ratio of the number of simulated floaters over the number of floaters in equilibrium is a proxy for the increase in competition due to the island dynamics. A ratio above 1 indicates stronger competition than under stable equilibrium conditions and a ratio below 1 indicates weaker competition than under stable equilibrium conditions. Competition is relatively high if the island size rapidly decreases while competition is relatively low if island size rapidly increases. Size changes of Rodrigues have a stronger effect on competition compared to size changes of Mauritius.

In our model, only 22.22% of the individuals of the population obtain a territory and breed, while the remainder of the individuals is so called floaters without a territory. The presence of floaters is very common in bird populations with distinct breeding territories and increases competition for resources.^43^ The number of floaters could therefore function as a measure of competitive pressure for territories. Our population model shows that the rapid coastal retraction would result in years with 3.14% more floaters present on Rodrigues compared to the expected number of floaters in equilibrium with a constant island size, but result in only 0.73% more floaters present on Mauritius compared to the expected number of floaters in equilibrium with a constant island size (Figure 4). This suggests that in years with rapid sea level rise competition for territories would temporarily increase more on Rodrigues than on Mauritius.

We hypothesize that this extreme rate of territory reduction during the solitaire lifespan acted as a powerful trigger for the emergence of intraspecific aggressive behavior, one that would not have acted as strongly on the population if coastal retraction rates acted on multigenerational timescales. The increase in competition due to displaced individuals from the progressive inland movement of the coastline was much larger on Rodrigues compared to Mauritius. Recurring extreme pulses of area contraction took place at the end of glacial periods on Rodrigues (Figure 3B) over the period from when the persistent volcanic island was formed 1.6 My ago,^25^ up to the end of the last ice age between 12 and 11 ky ago. Under gradual coastal contraction conditions, as experienced on Mauritius, the increase in territory competition would have been minor and easily absorbed over generational time. By contrast, we propose that the rapid decrease in island size in Rodrigues would have been conducive to aggressive trait development over the intense short pulses of area reduction, typically no more than a thousand years in duration.

### Fixation of aggressive traits

**Figure 5:**
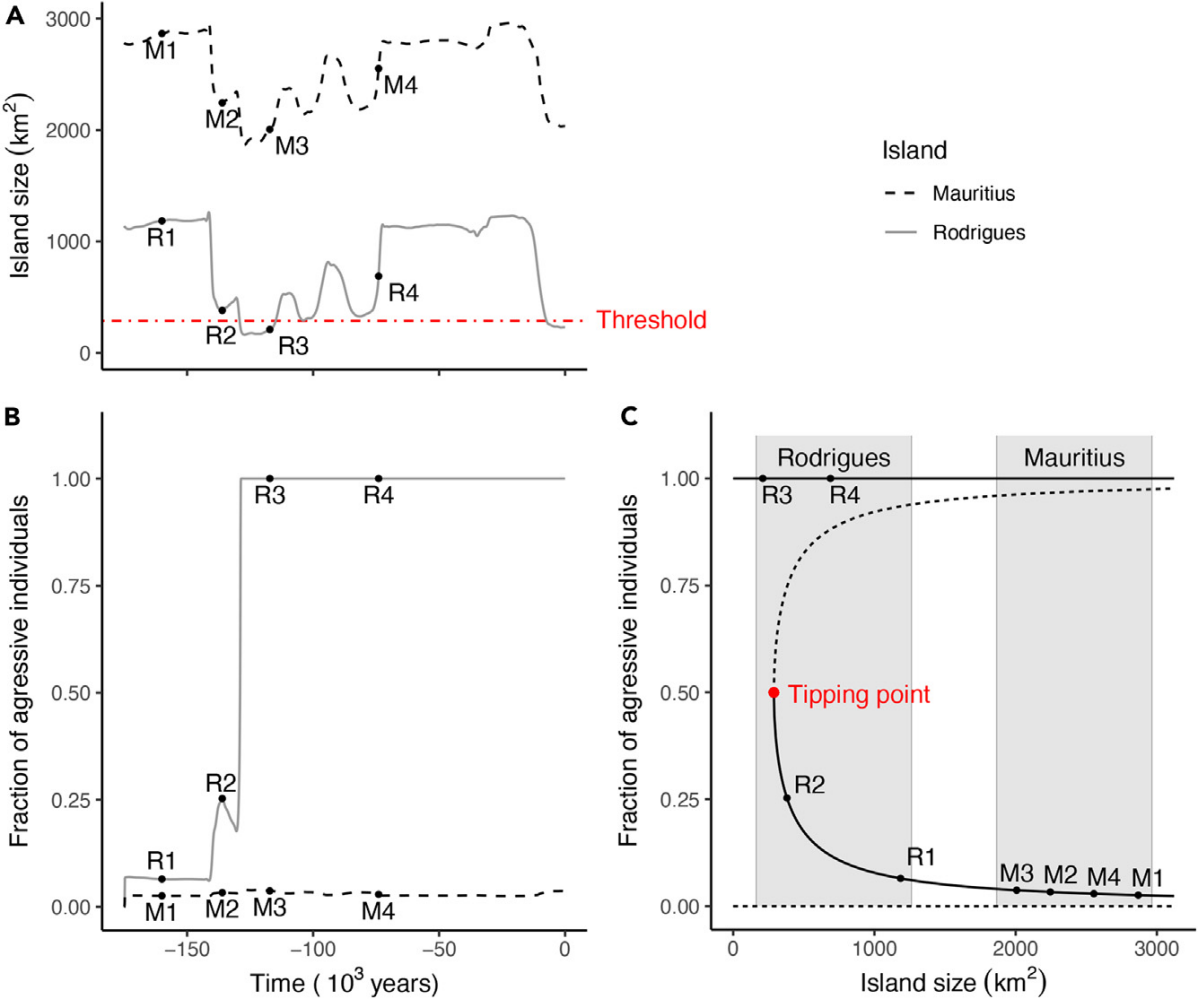
Dynamics of island size and population structure (A) Dynamics of the island size over time for Rodrigues (solid gray line) and Mauritius (dashed black line) calculated with the sea level model. To illustrate model dynamics, here a time window is presented in which the initiation time for the onset of sea level fluctuations was taken as a constant value from −175 ky until the last interglacial at −140 ky. The red dot-dashed line indicates the island size threshold below which fighters can take over the island, corresponding to the tipping point in figure c. (B) Dynamics of the fraction of aggressive individuals at Rodrigues (solid gray line) and Mauritius (dashed black line) calculated with the population model. Mauritius always maintains a low fraction of aggressive individuals, while Rodrigues switches to a state with only aggressive individuals as soon as the island size falls below a threshold value indicated with the red dot-dashed line in panel (A) and the tipping point in panel (C). (C) Bifurcation graph of the population model showing the fraction of aggressive individuals in equilibrium as a function of the island size. Solid lines indicate stable equilibria which are attractors of the dynamics and dashed lines represent unstable equilibria which are repellents of the dynamics. The two equilibria with a mixed strategy meet at a tipping point (red dot) and do not exist for a low island size, allowing the sudden shift to an island with only aggressive individuals. The tipping point indicated with a red dot corresponds to the threshold indicated with the red dot-dashed line in panel A. Gray areas indicate the size ranges within which the size of respectively Rodrigues and Mauritius fluctuate according to the sea level model. The tipping point lies within the territory size range of Rodrigues but not the territory range of Mauritius explaining the divergence in aggressive traits for the solitaire and the dodo. Labeled dots indicate recognizable points in the time dynamics to ease comparison between the panels.

We also used our model to examine the spread and dynamics of aggressive behavior in a population following its emergence. From the fossil record, we know that solitaires evolved aggressive phenotypic traits that became established throughout the entire population and in both sexes. We started simulations with a low number of aggressive individuals in a population of mainly non-aggressive individuals just before the start of an interglacial period, when island size is maximal (Figure 5A). Under these conditions, the simulations show that aggressive individuals on both Mauritius and Rodrigues stabilize at a small fraction of the population (Figure 5B). A small fraction of aggressive individuals will persist in the population because they always win a breeding territory when competing with non-aggressive individuals. Nonetheless, in our simulations, the fraction of aggressive individuals remains low when island size is maximal (Figures 5A and 5B); under maximal island size conditions the increased risk of injury associated with competition acts as a limit on the number of aggressive individuals in a population. Overall, our simulations show that most changes in island size resulted in only small changes in the fraction of aggressive individuals (Figures 5A and 5B).

However, we also found that when the island size decreased below a critical threshold size, the aggressive strategy became fixed in the population. At island sizes below the threshold the success of aggressive strategies switches from being marginally successful to progressively outcompeting the other strategies, up to the degree that the aggressive strategy would become fixed in the population (Figures 5A and 5B). The aggressive trait fixation scenario occurs abruptly and inevitably when the island size decreases below the threshold, a tipping point at which the risk of injury abruptly switches from being a strong limiting factor, to a stage where non-aggressive behavior is progressively outcompeted (Figures 5A–5C). As a result, non-aggressive individuals no longer get the opportunity to breed, and non-aggressive phenotypic traits disappear from the population. As both male and female solitaire exhibited extreme aggressive behavior when defending territories and rearing young, this strongly suggests the importance of protecting limited resources when breeding.^44^ Therefore, the fixation of an aggressive trait would have been an important evolutionary factor in solitaire behavior.

Of the two islands, only Rodrigues drops below the critical threshold due to the sea level induced island surface changes (Figures 5A–5C). Consequently, all newborn solitaires would be the offspring of individuals with an aggressive strategy once the threshold was reached, at which point non-aggressive individuals no longer had the opportunity to reproduce. This caused a tipping point in the dynamics of the population, eventually resulting in the fixation of aggressive behavior, because non-aggressive individuals would have been lost from the population (Figure 5C). Once aggressiveness was fixed in the population, a return of the island to a larger size would not result in a resurgence of non-aggressive behavior, because non-aggressive traits would have disappeared from the existing population (Figures 5A–5C). In Mauritius, our simulation model results indicate that the fraction of aggressive dodo individuals would have remained low throughout the island’s entire geological history, as its area contraction was less severe, which suggests that the dodo population never exceeded the tipping point (Figures 5A–5C).

In our model, non-aggressive and aggressive behavior is modeled as a pure strategy, which means that an individual will always use the same strategy and that the strategy is inherited by the offspring. Using this assumption, we tracked the fraction of the population displaying aggressive behavior. Another way the system could work, is that individuals have a mixed strategy, in which they display aggressive and non-aggressive behavior with a certain probability. This probability might shift due to evolutionary dynamics. In the classic Hawk-Dove game, the probability of the model with pure strategies and the model with mixed strategies both display the same results. The probability of displaying aggressive behavior in the model with mixed strategies is the same as the fraction of aggressive individuals in the model with the pure strategies.^8^^,^^9^ Overall, our model demonstrates mathematically that aggressive behavior might become fixed in an island population if the island size and therewith the resources fluctuate substantially. In our model, island size does not directly determine the behavior of individuals and the fixation of aggressive behavior is caused by the ecological dynamics of the population. More specifically, aggressive individuals are able to outcompete non-aggressive individuals only when the island size drops below a critical threshold, because they are able to occupy all territories. Even if non-aggressive behavior would emerge again in the population later, it would not spread through the population, because non-aggressive individuals cannot successfully hold a territory when competing with only aggressive individuals.

Although aggression may result from a complex array of factors involving demographic dynamics, habitat conditions, climatic, and seasonal conditions,^2^^,^^3^^,^^4^^,^^5^^,^^6^^,^^7^ the dodo and solitaire provide us with a novel working hypothesis and workflow to study the effects of area reduction under similar climatic conditions in comparable ecosystems. From our model we can predict that endemic birds which inhabit small islands (<100 km^2^) with a comparable tipping point history may exhibit comparable aggressive trait evolution. For instance, a species that could be of interest for testing our hypotheses is the notoriously aggressive Hood mockingbird on the Galapagos Island of Española. Our sea level model shows that this volcanic island also experienced extreme area reduction due to rapid sea level rise. As a result of this, the Hood mockingbird may have also reached a tipping point in aggressiveness. We hope that our work can contribute to the emerging field of eco-evolutionary dynamics by integrating a demographic model that examines trait selection under changing environmental conditions on sub-evolutionary timescales (<10ky) to explain behavior and phenotypic responses in species.^45^

### Conclusions

The integration of a Hawk-Dove game in a population model, in combination with fossil and geophysical data, shows that differences in land area change can explain the divergence in evolutionary strategies between two closely related, highly specialized endemic bird lineages. Repeated pulses of extremely rapid island size reduction were likely instrumental for the emergence of aggressive traits in the solitaire population, because they resulted in periodic strong increases in competition for resources. We demonstrated that extreme area contractions as recorded on Rodrigues are very rare and that most volcanic islands contract less. On Mauritius island size did typically not decrease so profoundly and consequently dodo did not experience comparable strong increases in resource competition.

Our model shows that aggressive behavior would become fixed in the population if the island size drops below a specific threshold. Although the exact threshold size at which the trait becomes fixed might depend on the parameter estimates, the general occurrence of the threshold is very robust in the model. The decline in island surface area and, as a result, the number of territories, appears to be sufficient to explain the fixation of aggressive behavioral traits and associated anatomy in the solitaire, contrasted with the absence of similar aggressive traits in the dodo. Moreover, our dodo-solitaire model with an adapted Hawk-Dove game for territory competition may serve as a wider mechanistic explanation of sudden shifts and fixation of aggressive character traits in nature, potentially paralleled in other organizational structures, such as businesses and governments.^41^

### Limitations of the study

The model in this study is developed to demonstrate that fluctuations in island size could lead to differences in aggressiveness between islands. As such, the model is only suitable for studying qualitative patterns as was done in this study, but it is not suitable for quantitative analysis and prediction. To make accurate quantitative predictions, the model should be tailored to a specific species using a detailed description of the life history of a species and very accurate parameter estimates are required, which are not available for the dodo and solitaire. In contrast, when studying qualitative patterns, the lack of accurate parameter estimates can be overcome by using bifurcation analysis to demonstrate the generality of patterns (see also STAR Methods and Figures S1 and S2).

Regardless of the type of the model, it is important to keep in mind that the model only accounts for the processes that are included in the model. The model analysis in this study is therefore very suitable to show that changes in island size could lead to divergence in aggressiveness, but this does not imply that this is the only mechanism that could lead to this pattern.

## Resource availability

### Lead contact

Further information and requests for resources should be directed to and will be fulfilled by the lead contact, Kenneth Rijsdijk (k.f.rijsdijk@uva.nl).

### Materials availability

#### Skeletal materials

Skeletal remains of the dodo (*Raphus cucullatus*) and the Rodrigues solitaire (*Pezophaps solitaria*) referenced in this paper were studied at the Natural History Museum (London and Tring; NHMUK) and the Royal College of Surgeons, London, UK (RCSHM). Also examined were dodo, solitaire and other columbiform skeletal materials in the collections of the Oxford University Museum of Natural History, Oxford, UK; University Museum of Zoology, Cambridge, UK; Musée National d’Histoire Naturelle, Paris, France; Naturalis Biodiversity Center, Leiden, The Netherlands; Durban Natural Science Museum, Durban, South Africa; Zoological Museum, University of Copenhagen, Copenhagen, Denmark; Museum of Comparative Zoology, Harvard, Cambridge, MA, USA; Yale Peabody Museum of Natural History, New Haven, CT, USA; the Mauritius Institute, Port Louis, Mauritius; and the DRP excavations (2005–2011) of the Mare aux Songes.

### Data and code availability

All sea level modeling input and output datasets, R script, and animations have been deposited at Figshare and are publicly available as of the date of publication. DOIs are listed in the key resources table.

• Data:

The Figshare data-directory hosts the computed area curves (area-directory) and input and output datasets which were used to compute paleo coastline configurations (reconstruct-directory). Paleo coastline configurations are provided in the mascarenes_st_curve-directory containing 53 coastline vector (vect.gpkg) and 53 raster maps (recrast-directory) for the extent of Rodrigues and Mauritius for the period over the past 26 ky, with time steps of 500 years including also the present day coastline.

• Code:

The Figshare code-directory hosts the R scripts and html files including (1) the calculations of area curves for Rodrigues and Mauritius for the last 3 My and 26ky based on respectively Eustatic Sea Level Curves (Lambeck, Cutler and Bintanja) and a Geophysical model (area_curve_calc.html), (2) visualizations of those area curves (area_curve_plot.html) and (3) a comparison with area curves of other islands across the world (area_curve_plot_comparison.html).

The R script for the population dynamics model is included as the file population_model.R.

• Other items:

An HTML animation of the modeled coastline configurations over time (mascarenes_st_curve.html).

## Acknowledgments

We thank the Natural History Museum, London, and Royal College of Surgeons, Oxford University Museum of Natural History, Oxford, UK; University Museum of Zoology, Cambridge, UK; Musée National d’Histoire Naturelle, Paris, France; Naturalis Biodiversity Center, Leiden, The Netherlands; Durban Natural Science Museum, Durban, South Africa; Zoological Museum, University of Copenhagen, Copenhagen, Denmark; Museum of Comparative Zoology, Harvard, Cambridge, MA, USA; Yale Peabody Museum of Natural History, New Haven, CT, USA; the Mauritius Institute, Port Louis, Mauritius; and the DRP excavations (2005–2011) of the Mare aux Songes for specimen access. We thank C. Davis, D. Kissling and M. Reindersma for discussions.

Funding: 10.13039/100006363National Geographic grant 9899-16 (L.P.A.M.C., J.P.H., A.J., and K.F.R.) French Laboratory of Excellence project TULIP grant ANR--10--LABX--41 (R.A.).

## Author contributions

Conceptualization: K.F.R., L.P.A.M.C., and J.C.C.; anatomical study: J.P.H., A.J., and L.P.A.M.C.; sea level modeling: K.F.R. and J.D.G.; population modeling: J.C.C.; discussion of population and ecological modeling: J.C.C., K.F.R., L.P.A.M.C., R.A., B.H.W., R.A., R.K., J.P.H., J.D.G., M.S., and A.J.; writing—original draft: L.P.A.M.C., K.F.R., and J.C.C. writing—review and editing: L.P.A.M.C., K.F.R., J.C.C., J.P.H., B.H.W., R.A., R.K., J.D.G., M.S., and A.J.

## Declaration of interests

Authors declare that they have no competing interests.

## STAR★Methods

### Key resources table

**Table:** 

REAGENT or RESOURCE	SOURCE	IDENTIFIER
**Software and algorithms**		

Population dynamics model R script (population_model.R)	This study	https://doi.org/10.21942/uva.26776705
Area curves Calculations for Rodrigues and Mauritius for the last 3 My and 26ky (area_curve_calc.html)	This study	https://doi.org/10.21942/uva.26776705
Area curves visualisations (area_curve_plot.html)	This study	https://doi.org/10.21942/uva.26776705
Comparison with area curves of other islands across the world (area_curve_plot_comparison.html). The data-directory hosts the computed area curves (area-directory) and input and output datasets which were used to compute paleo coastline configurations (reconstruct-directory). Paleo coastline configurations are provided in the mascarenes_st_curve-directory containing 53 coastline vector (vect.gpkg) and 53 raster maps (recrast-directory) for the extent of Rodrigues and Mauritius for the period over the past 26 ky, with time steps of 500 years including also the present day coastline.	This study	https://doi.org/10.21942/uva.26776705
HTML animation of modeled coastline configurations over time (mascarenes_st_curve.html).	This study	https://doi.org/10.21942/uva.26776705

**Other**		

GEBCO_2019 Grid	GEBCO Compilation Group (2019)^46^	https://doi.org/10.5285/836f016a-33be-6ddc-e053-6c86abc0788e
GEBCO_2021 Grid	GEBCO Compilation Group (2021)^47^	https://doi.org/10.5285/c6612cbe-50b3-0cff-e053-6c86abc09f8f
GEBCO_2023 Grid	GEBCO Compilation Group (2023)^48^	https://doi.org/10.5285/f98b053b-0cbc-6c23-e053-6c86abc0af7b

### Experimental model and study participant details

#### Anatomical observations

Anatomical information on the extinct Rodrigues solitaire (*Pezophaps solitaria*) and dodo (*Raphus cucullatus*) was derived from fossil specimens. Only a single complete associated skeletal specimen exists for the dodo, the Port Louis Thirioux specimen^21^ (Figure 2A). Disarticulated dodo remains are more abundant.^20^^,^^21^ For the Rodrigues solitaire, multiple associated skeletal specimens are known, including male and female specimens (Figures 2B and 2C), as well as disarticulated skeletal remains.^22^ It can be difficult to determine sex based on individual skeletal elements, but determination of sex was not important for anatomical behavioral analysis in this study, because both males and females of the Rodrigues solitaire possess skeletal wing weaponry. In the dodo, neither males nor females evolved skeletal wing weaponry. Data on healed fractures in the wing skeleton were derived from the NHMUK collections, bulk registration numbers NHMUK u/r and NHMUK A1441.

#### Mathematical model

The model we developed is a theoretical mathematical model, without any participants or experimental approach. Parameter values are tailored toward the dodo and solitaire on Mauritius and Rodrigues. Sex and gender do not affect the results as they are not a part of the theoretical model formulation.

### Method details

#### Sea level island area modeling

To investigate the area changes of the islands over time, we calculated the corresponding areas of the islands Mauritius and Rodrigues at 500 or 1000 years time steps, using a coastline reconstruction model.^26^^,^^31^ We calculated area changes for these islands for a single glacial interglacial period, to produce input for the demographic Hawk-Dove model to simulate the process of aggression initiation and its persistence in solitaires.

#### Eustatic sea level modeling

We used the last glacial-interglacial cycle (115 ky to present) as a representative example of an glacial-interglacial cycle. To model area change during this cycle we used an eustatic sea level model approach, whereby we assume that sea levels change globally at the same rate and magnitude (cf. Norder et al. 2018^31^). For this time window, we used the sea level curve reconstructed based on radiometric dated corals using 1000 years time steps.^49^^,^^50^ In reality, this curve does not provide a complete match due to geophysical differences over the globe. However, in practice we expect that the results of this modeling approach deviates only to small degree from the geophysical modeling as the Mascarene islands are in a far field area and sea levels here followed the eustatic global response.^51^

#### Geophysical sea level modeling

As the rates of coastal contractions are highest both in magnitude and velocity at the termination of the ice ages, we specifically calculated the effects of 130 m of sea level rise on rates of area loss and coastline retreat over this period from 26 ky to present. For the period of fastest sea level rise between 12 and 11 ky ago we measured the maximum coastline retreat using Euclidean distances from the coastline positions of the two subsequent time steps. For this period we used a geophysical based model as the geophysical data are resolved for this period.^26^ For our insular surface reconstructions we used an output resolution of 0.0333 ° × 0.0333 ° (∼500 m × 500 m) as derived from the global integrated topographic and bathymetric raster provided by General Bathymetric Chart of Oceans (GEBCO Compilation Group, 2019, 2021, 2023).^46^^,^^47^^,^^48^

Sea level modeling input and output datasets, R script, and animation, are available in the Supplemental Information.

#### Population dynamics modeling

##### Model with only non-aggressive individuals

We first model the dynamics of a population without aggressive behavior to explore whether the change in island size of Mauritius and Rodrigues change the competition for territories in the dodo and solitaire population. The dynamics of the number of individuals in the next year (N[t+1]) depends on the birth term (B[t]) and the per capita death rate (μ) in the current year:

(Equation 1) N[t+1]=B[t]+(1−μ)N[t]

Offspring production is either limited by the number of territories (K[t]) if the population is large or the number if individuals (N[t]) if the population is small. We assume that offspring mature and survive to the following year with a fixed success rate (*b*).

(Equation 2) $$B\left[t\right]=\left\{ \begin{array}{c} bK\left[t\right],\;N\left[t\right]>K\left[t\right]\\ bN\left[t\right],\;N\left[t\right]\leq K\left[t\right] \end{array}\right.$$

From these equations it is clear that a small population (N<K) will grow over time if (b>μ). Eventually the population will become bigger and reaches a stable equilibrium ($N^{*}$) determined by the number of territories (*K*), the reproductive success (*b*) and the mortality rate (μ):

(Equation 3) $$N^{*}=\frac{bK}{\mu }$$

From the whole population, only *K* individuals obtain a territory and breed, while the remainder of the individuals are floaters without a territory. As floaters provide the competition for territories, we can use the fraction (or percentage) of floaters in the population as the measure of competition, in which a higher fraction of floaters indicates more competition.

Changes in the number of territories, due to the changes in island size, might result in transient dynamics with higher competitive pressure. To express the change in competitive pressure due to changes in island size, we simulate the number of individuals using (Equation 1), (Equation 2) and compare the fraction of floaters in the simulations to the fraction of floaters in equilibrium (Figure 4). The number of territories for the simulations is directly calculated from the island sizes modeled in Figure 4 by assuming every territory covers an area of 0.104 km^2^. The mortality rate (μ) is set to 1/30, which corresponds to an average lifespan of 30 years. The chick survival is unknown and is set to 0.15. Because our model only accounts for one sex, this can be interpreted as a chick survival of 0.3 in a population with an equal sex ratio.

##### Model with non-aggressive and aggressive individuals

To model the dynamics of aggressiveness in the population we extend the model to explicitly model the number of aggressive and non-aggressive individuals ($N_{A}$ and $N_{F}$ respectively).

(Equation 4) $$N_{A}\left[t+1\right]=B_{A}\left[t\right]+\left(1-\mu \right)N_{A}\left[t\right]$$

(Equation 5) $$N_{F}\left[t+1\right]=B_{F}\left[t\right]+\left(1-\mu \right)N_{F}\left[t\right]$$

Competition affects the division of territories and consequently affects the birth term in two ways.

Firstly, competition for territories follows a classic Hawk-Dove game with the following payoff matrix, representing the probability to win the fight:

(Equation 6) $$P=\left[\begin{array}{ccc} & A & F\\ A & 0.5 & 1\\ F & 0 & 0.5 \end{array}\right]$$

This matrix does not include the additional costs for aggressive behavior because it is included separately as suggested by Argasinski and Broom (2013).^41^ The fraction of territories obtained by aggressive and non-aggressive individuals ($p_{A}$ and $p_{F}$ respectively) can be derived from this payoff matrix using classic game theory.^8^^,^^9^

(Equation 7) $$p_{A}=\frac{\left(0.5\left(N_{A}-1\right)+N_{F}\right)N_{A}}{\left(0.5\left(N_{A}-1\right)+N_{F}\right)N_{A}+0.5\left(N_{F}-1\right)N_{F}}$$

(Equation 8) $$p_{F}=\frac{0.5\left(N_{F}-1\right)N_{F}}{\left(0.5\left(N_{A}-1\right)+N_{F}\right)N_{A}+0.5\left(N_{F}-1\right)N_{F}}$$

Secondly, an aggressive individual may encounter another aggressive individual, they may fight and they may be wounded to the point not to be able to breed in the current year. We computed the number of aggressive individuals that cannot breed as $c\left(N_{A}-1\right)N_{A}$, where *c* scales the injury risk. Wounded individuals cannot breed in the current year and therefore have to be subtracted from the breeding population of aggressive individuals ($N_{A}-c\left(N_{A}-1\right)N_{A}$). Similar to the model described above for only non-aggressive individuals, offspring reproduction is limited by the non-wounded breeding population if the population is small and by the number of territories if the population is large. Based on these adaptations we can reformulate the birth terms for aggressive and non-aggressive individuals:

(Equation 9) $$B_{A}\left[t\right]=\left\{ \begin{array}{c} bp_{A}K\left[t\right],\;p_{A}K\left[t\right]<N_{A}\left[t\right]-c\left(N_{A}\left[t\right]-1\right)N_{A}\left[t\right]\\ b\left(N_{A}\left[t\right]-c\left(N_{A}\left[t\right]-1\right)N_{A}\left[t\right]\right),\;p_{A}K\left[t\right]\geq N_{A}\left[t\right]-c\left(N_{A}\left[t\right]-1\right)N_{A}\left[t\right] \end{array}\right.$$

(Equation 10) $$B_{F}\left[t\right]=\left\{ \begin{array}{c} bp_{F}K\left[t\right],\;p_{F}K\left[t\right]<N_{F}\left[t\right]\\ bN_{F}\left[t\right],\;p_{F}K\left[t\right]\geq N_{F}\left[t\right] \end{array}\right.$$

These equations are used to simulate the number of non-aggressive and aggressive individuals on Rodrigues and Mauritius. The parameter settings are the same as for the model in the population dynamics model with only non-aggressive individuals and the scalar for the injury risk (*c*) is set to the arbitrary value of 0.00025, because it cannot be estimated. These simulations result in Figure 4 in the main article. The impact of the parameter values is discussed in the last paragraph and Figure S1.

##### Bifurcation analysis

To show that the presence of a tipping point is driving the divergent dynamics of aggressiveness we study the equilibria as a function of the number of territories. The model presented above results in four equilibria for the fraction of aggressive individuals ($f_{A}=\frac{N_{A}^{*}}{N_{A}^{*}+N_{F}^{*}}$), of which one has only aggressive individuals, one has only non-aggressive individuals and two have a mixed strategy (Figure 5C):

(Equation 11) $$f_{A}=0$$

(Equation 12) $$f_{A}=\frac{2\left(1+\frac{b-\mu }{bc}\right)}{\left(\frac{bK}{\mu }+1\right)\pm \sqrt{{\left(\frac{bK}{\mu }-1\right)}^{2}-4\frac{bK}{\mu }\left(1+\frac{b-\mu }{bc}\right)}}$$

(Equation 13) $$f_{A}=1$$

The equilibrium with only aggressive individuals and one of the equilibria with a mixed strategy are stable and act as attractors of the dynamics. The equilibrium with only non-aggressive individuals and the other equilibrium with a mixed strategy are repeller of the dynamics. The two equilibria with mixed strategies meet at a limit point or tipping point and do not exist for low number of territories. At the start of the simulations presented in the article, the dynamics lies around the stable equilibrium with a mixed strategy. When the size of Rodrigues decreases below the tipping point, the individuals on the island change to an entirely aggressive strategy. Due to the tipping point and the presence of the unstable equilibria, the dynamics of Rodrigues cannot move back to the stable equilibrium with a mixed strategy. The size of Mauritius does never decrease below the tipping point and the dynamics on this island therefore stays around the stable equilibrium with a mixed strategy (Figure 5C).

The location of the tipping point can be derived from Equation 12:

(Equation 14) $${\left(\frac{bK}{\mu }-1\right)}^{2}-4\frac{bK}{\mu }\left(1+\frac{b-\mu }{bc}\right)=0$$

The island area at which the tipping point occurs depends on the birth rate (*b*), the scalar for the injury risk of aggressive individuals (*c*) and the mortality rate (μ). The values of these parameters are very difficult to estimate from historical data. Figure S1 shows how the values of these parameters affect the island size at which the tipping point occurs. It is important to keep in mind that simultaneous changes in multiple parameters might have additional effect (Figure S2). Nonetheless, in Figure S2 we show that the parameter range in which the tipping point will occur at Rodrigues is much larger than the parameter range in which the tipping point occurs at Mauritius. This demonstrates that the switch into a population comprising of aggressive individuals is likely to occur at Rodrigues and unlikely to occur on Mauritius.

### Quantification and statistical analysis

The theoretical mathematical model is formulated with ordinary differential equations (ODEs). The complete equations of the models are explained in the method details section. The theoretical population model is analyzed through numerical simulations and mathematical bifurcation analysis in R.

In Table 1, the number of healed fractured wing bones observed in Rodrigues solitaire fossils is given as a fraction of the total number of skeletal elements examined. The numbers represent individual skeletal elements and are reported separately for the right and left side.
