# Supplementary material for: Sea level rise and the evolution of aggression on islands
Source: iScience. 2024 Oct 22;27(11):111236. doi: 10.1016/j.isci.2024.111236 (PMC11574790; doi:10.1016/j.isci.2024.111236)
Supplement: Document S1. Figures S1 and S2 [file mmc1.pdf]

iScience, Volume *27*

Supplemental information

Sea level rise and the evolution

# of aggression on islands

Kenneth F. Rijsdijk, Jasper C. Croll, Julian P. Hume, Anwar Janoo, Robin Aguilée, Johannes De Groeve, Rosemarie Kentie, Menno Schilthuizen, Ben H. Warren, and Leon P.A.M. Claessens

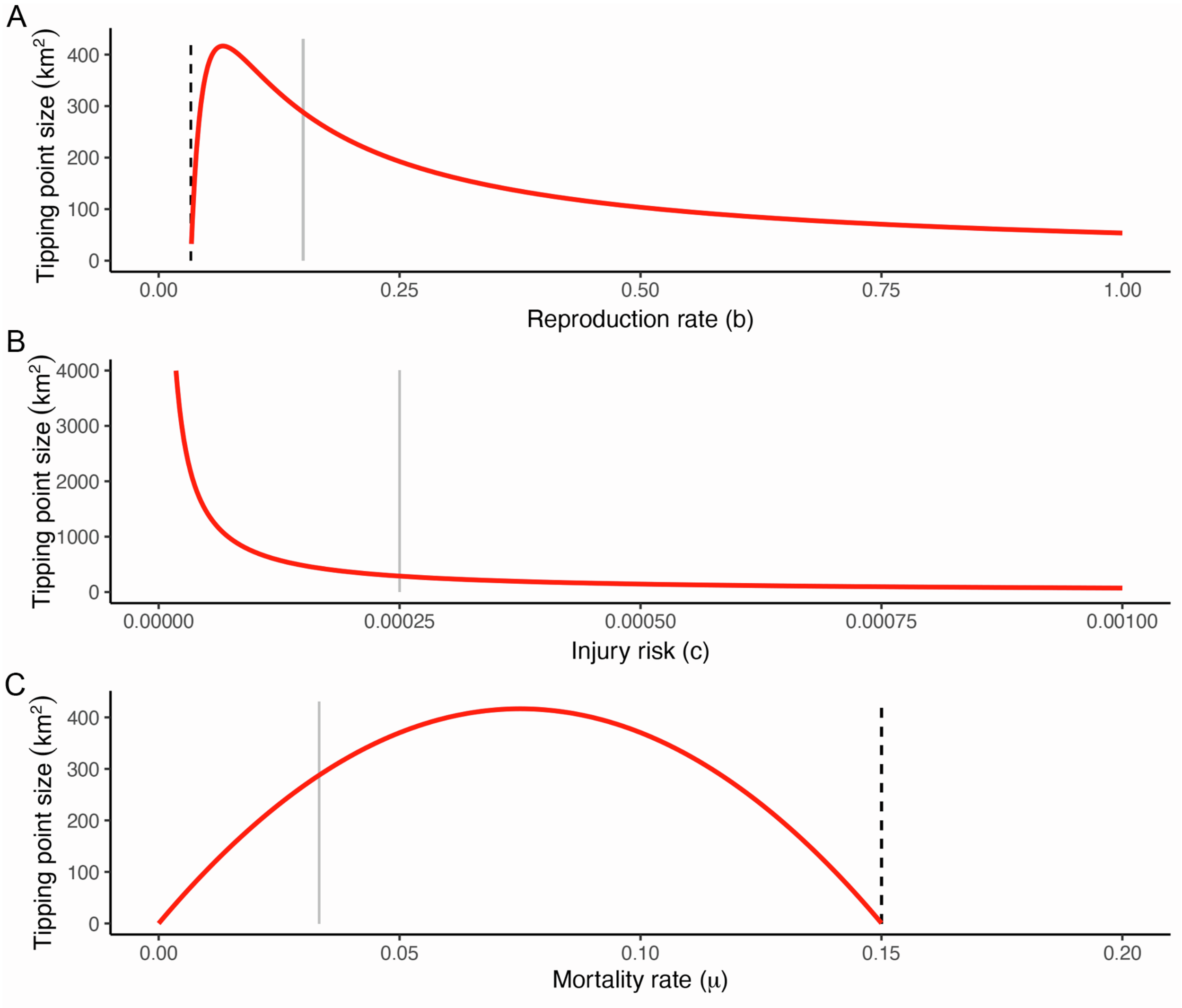

Figure S1. Effect of the birth rate (*a*) injury risk scalar (*b*) and mortality rate *(c)* on the island size at which the tipping point occurs. Related to STAR Methods. Red lines indicate the island size at which the tipping point occurs. dashed black lines indicate the extinction boundary. The population cannot persist at reproduction rates lower than the black dashed line in panel a. Similarly, the population cannot persist at mortality rates higher than the black dashed line in panel c. Grey solid lines indicate the parameter values used in the simulations.

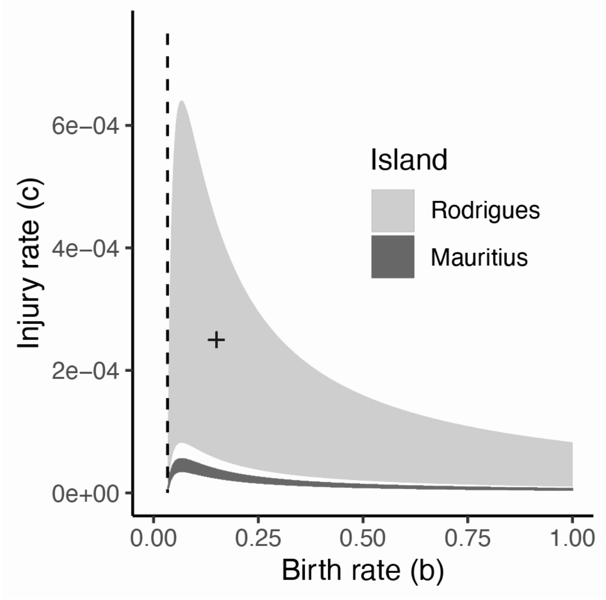

Figure S2. Parameter space of the birth rate (*b*) and injury risk scalar (*c*) at which the tipping point falls within the size range of Mauritius and Rodrigues. Related to STAR Methods. Below the light grey area, Rodrigues will always be entirely occupied by aggressive individuals. Within the light grey area, the island size dynamics of Rodrigues could cause a tipping towards only aggressive individuals. Above the light gray area, Rodrigues is likely to be occupied by a mix of aggressive and non-aggressive individuals. However large perturbations in the population due to other factors could cause the fixation of aggressive behaviour on Rodrigues. Similarly, below the dark grey area, Mauritius will always be entirely occupied by aggressive individuals. Within the dark grey area, the island size dynamics of Mauritius could cause a tipping towards only aggressive individuals. Above the dark grey area, Mauritius is likely to be occupied by a mix of aggressive and non-aggressive individuals. However very large perturbations in the population due to other factors could cause the fixation of aggressive behaviour on Mauritius. To the left of the black dotted line the birth rate is too low for the population to persist. The cross indicates the parameter settings used in the simulations.
